# Beyond Bacteria: The Emerging Role of the Gut Virome and Mycobiome in Celiac Disease

**DOI:** 10.3390/nu18162721

**Published:** 2026-08-20

**Authors:** Aurelio Seidita, Mirco Pistone, Federica Latteri, Francesca Di Stefano, Pasquale Mansueto, Sonia Giardina, Gabriele Spagnuolo, Alessandro Termini, Salvatore Maestri, Alessandra Giuliano, Salvatore Accomando, Carola Buscemi, Francesca Mandreucci, Antonio Carroccio

**Affiliations:** 1 Unit of Internal Medicine, “V. Cervello” Hospital, Ospedali Riuniti “Villa Sofia-Cervello”, 90146 Palermo, Italy; aurelio.seidita@unipa.it (A.S.); mirco.pistone@gmail.com (M.P.); francesca.distefano04@unipa.it (F.D.S.); soniagiardina2@gmail.com (S.G.); gabrielespagnuolo1995@gmail.com (G.S.); alessandrotermini02@gmail.com (A.T.); maestrisalvatore@gmail.com (S.M.); alegiuliano94@gmail.com (A.G.); carola.buscemi@gmail.com (C.B.); francesca.mandreucci@gmail.com (F.M.); 2 Department of Health Promotion Sciences, Maternal and Infant Care, Internal Medicine and Medical Specialties (PROMISE), University of Palermo, 90127 Palermo, Italy; pasquale.mansueto@unipa.it (P.M.); salvatore.accomando@unipa.it (S.A.); 3 Institute for Biomedical Research and Innovation (IRIB), National Research Council (CNR), 90133 Palermo, Italy; 4 Unit of Gastroenterology, “V. Cervello” Hospital, Ospedali Riuniti “Villa Sofia-Cervello”, 90146 Palermo, Italy; federicalatteri@gmail.com

**Keywords:** celiac disease, gut virome, mycobiome, microbiome, host-microbial interactions, gluten-free diet

## Abstract

**Background/Objectives:** Celiac disease (CeD) is a complex autoimmune enteropathy triggered by gluten intake in genetically predisposed individuals. While the bacterial microbiome has been extensively studied in CeD, the role of the gut virome and mycobiome is less well defined. Emerging evidence suggests that viruses and fungi may influence mucosal immunity, intestinal permeability and gluten tolerance, acting as potential environmental cofactors in the onset of this disease. **Methods:** A narrative review of the literature was conducted to summarize current knowledge of the composition, development and function of the gut virome and mycobiome and to explore their potential involvement in CeD pathogenesis. **Results:** The gut virome shows high interindividual variability and dynamic maturation early in life. Multiple studies have associated early *enterovirus* or *parechovirus* infections with an increased risk of celiac autoimmunity, supporting a virus–gluten “double hit” model. Viral dysbiosis in CeD has been reported to include an enrichment of *human polyomavirus 2 (JC polyomavirus)* and of specific enterobacterial phages. The mycobiome, although less abundant, also displays disease-associated alterations: cross-sectional mycobiome studies have reported higher relative abundance of *Saccharomyces*, *Candida*, and *Tricholomataceae* and lower relative abundance of *Pichia* and *Pneumocystis* taxa in children with CeD. **Conclusions:** Current evidence, although preliminary, supports associations between selected viral exposures and CeD-related immune alterations, whereas current phageome and mycobiome findings mainly describe cross-sectional compositional differences. These data do not establish temporal direction or causality, and further longitudinal and mechanistic investigations are required before preventive or therapeutic implications can be considered.

## 1. Introduction

Celiac disease (CeD) is a chronic, immune-mediated enteropathy determined by the ingestion of gluten-containing cereals in genetically susceptible individuals [1]. It affects approximately 1% of the global population, although prevalence varies according to geography, age, and diagnostic strategies [2]. The disease is characterized by a genetic predisposition (primarily the Human Leukocyte Antigens (HLA)-DQ2 and HLA-DQ8 haplotypes) combined with exposure to gluten, but these two factors alone do not fully explain disease onset. Indeed, only a minority of genetically predisposed individuals progress to the clinical disease, suggesting that additional environmental triggers play a fundamental role in shaping immune tolerance or intolerance to gluten [1,3,4].

Over the past two decades, the gut microbiome has emerged as a key environmental factor influencing immune system development, epithelial integrity, and oral tolerance. While most research has traditionally focused on bacterial communities [5,6,7], the human gut ecosystem also includes viruses (the virome), fungi (the mycobiome), archaea and protozoa, each contributing uniquely to host–microbe interactions [8,9,10]. These microbial components display dynamic changes throughout life, particularly during early childhood, a critical window for immune maturation [11,12,13]. Alterations in the bacterial microbiome have been linked to CeD pathogenesis through mechanisms such as dysbiosis, increased intestinal permeability, and modulation of inflammatory pathways [14].

Microbial dysbiosis, defined as an imbalance in the composition and function of the intestinal microbial ecosystem characterized by the loss of beneficial microorganisms, the expansion of pathobionts, and reduced microbial diversity, has been increasingly implicated in the pathogenesis of immune-mediated diseases, including celiac disease [15].

An increasing number of research groups have shifted their attention from the bacterial to the viral and fungal components of the gut microbiome, highlighting how the interaction between these different components and between them and other environmental factors (e.g., diet, antibiotic use, mode of birth, and weaning), may be the missing ‘added factor’ linking genetic predisposition, gluten exposure and the development of an autoimmune response [5,6,7,14,16,17].

The aim of this review is to summarize recent advances in the understanding of the gut virome and mycobiome in the pathogenesis and natural history of CeD, critically appraise the strengths and limitations of the available evidence, and identify priorities for future research. Particular attention is given to their potential contribution to disease mechanisms and to their possible value as future biomarkers or targets for further investigation, rather than established therapeutic strategies.

## 2. Materials and Methods

This study was designed as a narrative review supported by a structured, non-systematic literature search. The structured search was performed to improve transparency and coverage of the available literature; however, no systematic-review methodology, formal risk-of-bias assessment, or meta-analysis was planned.

A comprehensive search of the literature (last update 8 August 2026) was conducted across major biomedical databases, including PubMed, Scopus and Web of Science, without time restrictions and including the following research terms (further details in Supplementary Table S1):

1. (“celiac disease” OR “coeliac disease” OR celiac OR coeliac) AND (virome OR “gut virome” OR “intestinal virome” OR “viral microbiome” OR enterovirus* OR parechovirus* OR reovirus* OR bacteriophage* OR phage* OR phageome)
2. (“celiac disease” OR “coeliac disease” OR celiac OR coeliac) AND (mycobiome OR “gut mycobiome” OR “intestinal mycobiome” OR “fungal microbiome” OR mycobiota OR “fungal microbiota” OR “fungal community” OR “fungal communities” OR fungi OR fungal)

Pre-specified exclusion criteria were:

1. Articles published in languages other than English;
2. Duplicate records retrieved from multiple databases;
3. Articles with full texts unavailable;
4. Studies not addressing the research question or falling outside the scope of the review.

**Figure 1:**
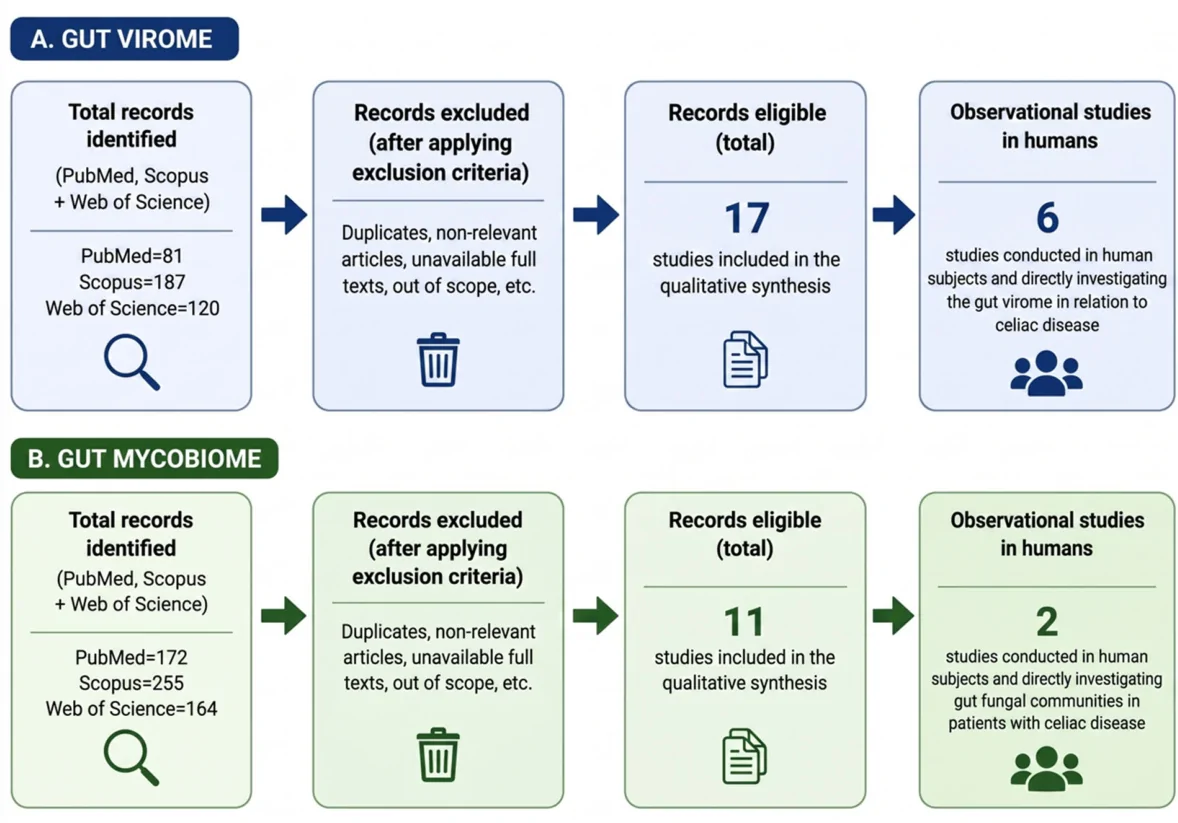
Schematic study-selection diagram for the structured literature search. (**A**): gut virome literature. (**B**): gut mycobiome literature. The diagram is provided for descriptive transparency and does not represent a PRISMA systematic-review flowchart.

The literature search on the gut virome identified a total of 388 articles. After applying the exclusion criteria, 17 studies met predefined eligibility criteria and were included in the qualitative synthesis. Among these, six were observational studies conducted in human subjects and directly investigated the gut virome in relation to CeD, whereas the remaining eligible publications comprised experimental and/or mechanistic studies providing complementary evidence on the potential role of viral communities in CeD (Figure 1A).

The search on the gut mycobiome identified 591 records. Following the removal of non-eligible articles, 11 records were included. Of these, only two were observational studies conducted in human subjects and directly investigated gut fungal communities in patients with CeD, whereas the remaining eligible studies provided complementary experimental and/or mechanistic evidence (Figure 1B).

Results from the database search were independently screened by 4 authors (AS, MP, FDS, FM), and the full texts of all the papers responding to the inclusion/exclusion criteria were reviewed. In the event of disagreement, the judgement of the study supervisor (AC) was considered final. Consistent with the narrative-review design, no validated risk-of-bias instrument, formal evidence-grading framework, scoring system, or predefined quantitative rubric was applied. Accordingly, no formal evidence-strength grading was performed.

To enhance the contextual completeness of the review, backward citation searching was also performed. Additional references identified through this process were used to support the general background and discussion but were not included in the disease-specific qualitative synthesis summarized in Figure 1 and Table 1 and Table 2.

## 3. From Colonization to Disease: The Gut Virome in the Celiac Context

It is estimated that the number of bacterial cells associated with the human body is roughly equal to the number of human cells, approximately 10^13^ in total. Virus-to-bacterium ratios in the human gut are highly variable and have been estimated to range from approximately 0.1 to 10 depending on the population studied, sequencing methods, and ecological conditions [26]. It has been known for years that the human intestine is colonized by multiple bacterial species in enormous quantities [27]. Less well known, however, is the presence of multiple viral strains in the intestine, which play a fundamental role in homeostasis, not only in the intestine but throughout the entire organism [28].

### 3.1. Composition of the Gut Virome

Viral populations colonizing the human body vary greatly depending on the anatomical site analyzed. The intestine, being the site with the highest concentration of bacterial cells and thus providing a wide variety of hosts for viral replication, is the organ that contains the most abundant viral populations, collectively known as the “gut virome” [26].

Viruses can be classified according to their genome (RNA, DNA, single- or double-stranded) or the structure of the viral capsid (spherical, filamentous, projectile, pleomorphic, tailed) [26]. In recent years, the development of sequencing techniques (“shotgun sequencing”) has made it possible to study the genomes of multiple viral strains which might be found both in the environment and in the stool samples of healthy and sick subjects, with the aim of identifying the association between “viral dysbiosis” and the onset/progression of several diseases [29]. The gut virome comprises two biologically distinct components: bacteriophages and eukaryotic viruses. Bacteriophages primarily influence the bacterial microbiome through predation and horizontal gene transfer, whereas eukaryotic enteric viruses directly interact with epithelial and immune cells. Consequently, their contribution to CeD pathogenesis and the interpretation of metagenomic studies should be considered separately [30,31].

More than 90% of the viruses belonging to the human virome are bacteriophages [32], including numerous tailed double strand DNA (dsDNA) phages currently classified within the class *Caudoviricetes*, as well as single strand DNA (ssDNA) phages of the family *Microviridae*. Earlier studies, using the historical morphology-based classification, described many early-life tailed phages as members of *Siphoviridae*, *Podoviridae*, and *Myoviridae*; these families and the former order *Caudovirales* have since been abolished and substantially reorganized within *Caudoviricetes*. The remainder, which might colonize archaea or human cells, are represented by either DNA viruses such as *Anelloviridae*, *Orthoherpesviridae* and *Adenoviridae*, or RNA viruses such as the family *Coronaviridae* and the genera *Sapovirus* and *Rotavirus* [33]. Some can cause acute infections, while others can establish long-term latency. Some viruses participate in benign colonization and cannot be associated with any specific disease, thus appearing as long-term “passengers” or “commensals”. In addition, a small percentage is represented by transient strains usually associated with food intake [29].

High interindividual variability in the human virome has been reported in many studies, related to both modifiable and non-modifiable conditions [34], such as age [33], birth mode [35], breastfeeding [36], fasting [37] and dietary intake (including malnutrition) [37,38,39,40,41,42], antibiotic therapy [43], as well as pathological conditions [44,45,46,47,48]. However, in a healthy adult, the human virome is usually relatively stable over time, paralleling the stability of the gut microbiome [34]. Given its extensive interconnectivity with all living cells, particularly in relation to other components of the microbiome, the virome cannot be studied in isolation; the gut virome and bacterial microbiome follow similar trajectories, engaging in reciprocal interactions in both health and disease [49]. Although efforts have been made to design specific tools for assembling viral sequences, these still need to be actively developed, in contrast with those of their bacterial counterparts. Indeed, reconstructing viral genomes is far more challenging due to several inherent complexities: viral taxonomy is not based on clear virus species definitions, many viral life cycles are only partially understood, sequencing reads often align to multiple related genomes, and eukaryotic genomes harbor numerous embedded viral remnants [50]. Furthermore, a substantial fraction of sequences continues to fall within the poorly characterized so-called ‘viral dark matter’ category: preliminary metagenomic studies indicate that the majority of viral genome sequences cannot be matched to any known viral reference. This phenomenon highlights a significant gap in our understanding of the taxonomic composition and structure of the phageome in the gut and poses a major challenge for bioinformatic analysis [51].

### 3.2. Viral Intestinal Colonization

A study by Breitbart et al. analyzed viral genomes in the meconium of infants just after birth, showing no viral populations. In contrast, in the same study, a high concentration of viruses was found in the stools of one-week-old infants, suggesting that infants did not have a virome at birth, but were colonized immediately afterward [52].

These preliminary findings were then supported by broader metagenomic surveys, which showed that gut virome emerges following the first exposure to maternal, environmental, and dietary microbial communities. A metagenomic analysis compared fecal virome samples of 1-year-old infants born by vaginal delivery with those born by cesarean section; the birth mode impacted the gut virome, with a greater viral heterogeneity in infants born by vaginal delivery [35]. The first colonizing viruses are mostly bacteriophages belonging to the *Siphoviridae*, *Podoviridae*, and *Myoviridae* families, which mirror the early bacterial communities of the neonatal gut. These family names follow the historical International Committee on Taxonomy of Viruses (ICTV) classification commonly adopted in earlier microbiome studies; recent ICTV taxonomy has substantially reorganized tailed bacteriophages. These pioneer phages are believed to shape bacterial succession through density-dependent predation and modulation of horizontal gene transfer. Further work has shown that temperate phages, often acquired from maternal sources or hospital environments, dominate at first, providing a “seed” virome through prophage induction. Over time, this community shifts toward greater lytic activity as bacterial hosts expand, and eukaryotic viruses begin to appear as infants encounter dietary and environmental exposures [53].

In addition to birth mode, other factors influence the establishment and maturation of the virome, such as breastfeeding; this is associated with an increased number of *Bifidobacterium*-infecting phages and with a reduction in potentially pathogenic eukaryotic viruses, likely due to the antiviral and microbiome-modulating components of human milk. Moreover, dietary transitions, such as the introduction of solid foods, dramatically reshape both bacterial and viral communities, increasing the diversity and metabolic potential of the virome. Host genetics, immune development, antimicrobial exposure, and environmental interactions (siblings, pets, daycare, etc.) further contribute to inter-individual variability [54].

Further studies have emphasized that the infant virome develops along a predictable trajectory: it is first composed of populations of low-complexity phages, followed by a gradual enrichment through childhood, which reflects the maturation of the underlying bacterial microbiome. These dynamics appear to be critical for immune education and metabolic programming, suggesting that early perturbations of the virome, such as from drug intake or cesarean section, may influence subsequent susceptibility to inflammatory and metabolic diseases [55].

### 3.3. Virome in Health and Disease

The gut virome contributes to intestinal homeostasis through continuous interactions with bacterial communities and the host immune system. Bacteriophages regulate bacterial population dynamics, horizontal gene transfer, and microbial diversity, thereby indirectly influencing epithelial barrier integrity and immune maturation. In healthy individuals, these interactions help maintain microbial stability, whereas virome alterations may promote dysbiosis, immune dysregulation, and chronic intestinal inflammation [40,53,56].

Growing evidence suggests that virome alterations are associated with several immune-mediated disorders [57], including inflammatory bowel disease [58,59] and type 1 diabetes mellitus [60,61]. However, current evidence remains largely associative, and the mechanisms linking virome alterations to disease onset are still poorly understood. These observations provide the biological rationale for investigating the contribution of the gut virome to celiac disease.

### 3.4. Virome and CeD

Evidence linking viral exposures and the gut virome to CeD derives from substantially different study designs and should therefore be interpreted according to the type of evidence available. Experimental studies primarily provide mechanistic support for virus-mediated loss of oral tolerance; prospective and longitudinal human studies address the temporal relationship between enteric viral exposure and subsequent CeD autoimmunity or disease, whereas cross-sectional virome and phageome studies identify disease-associated compositional differences without establishing temporal direction or causality. These different lines of evidence are therefore discussed separately below.

#### 3.4.1. Experimental and Mechanistic Evidence

Viral infections may contribute to the pathogenesis of CeD by disrupting oral tolerance to dietary antigens [62,63]. Common viral agents, such as *reovirus* or *enterovirus*, have been found to trigger inflammatory reactions against gluten components, favoring T Helper 1 (Th1) immune responses over Regulatory T cells (Tregs). Furthermore, in murine models type I interferon (IFN) production during viral infections disrupted oral tolerance and promoted the development of CeD [62]. Subsequent mechanistic studies further clarified this pathway. Brigleb et al. demonstrated that intestinal infection with *reovirus* strain T1L recruits and activates natural killer (NK) cells in mesenteric lymph nodes in a type I IFN-dependent manner. Activated NK cells contribute to type II IFN signaling and promote inflammatory dendritic-cell and T-cell responses, whereas NK-cell depletion impairs virus-induced loss of oral tolerance [64]. Interestingly, virus-mediated disruption of oral tolerance may also be influenced by other members of the intestinal ecosystem. In a murine model, Medina Sanchez et al. showed that the gut protist *Tritrichomonas arnold* protected against *reovirus*- and murine *norovirus*-mediated loss of oral tolerance by restraining the pro-inflammatory program of dietary antigen-presenting dendritic cells, limiting Th1 responses and promoting Tregs responses [65]. These findings suggest that the effect of enteric viruses on dietary-antigen tolerance may depend not only on direct virus–host interactions but also on interkingdom interactions within the intestinal microbial ecosystem.

Indirect observations in humans also support the biological plausibility of virus-related immune activation; for example, interferon therapy for hepatitis C virus has historically been associated with CeD occurrence or recognition in susceptible individuals [66]. However, such observations should not be considered equivalent to prospective evidence linking naturally occurring enteric viral infections to subsequent CeD.

#### 3.4.2. Prospective and Longitudinal Human Evidence on *Enterovirus* and *Parechovirus* Exposure

Among the available human evidence, prospective and longitudinal cohort studies are particularly informative because viral exposure is assessed before the development of CeD autoimmunity or clinical disease. Several studies have focused specifically on *enterovirus* and *parechovirus* infections during early life.

In 2021, a study conducted in Norway by Tapia et al. showed that *parechovirus* infections in early childhood were associated with the development of CeD in genetically at-risk children [67]. Conversely, according to the study conducted by Carlsson et al., *enterovirus* infection during pregnancy was not associated with the development of CeD in childhood [68].

The most robust contribution to understanding the CeD-virome relationship came from the TEDDY study, which analyzed the fecal viral component via metagenomics. This study demonstrated that cumulative exposure to *enteroviruses* during the first two years of life was associated with an increased risk of CeD autoimmunity, and an enhanced effect was observed in cases of higher gluten consumption, suggesting a virus–gluten “double hit” model [18]. Similar results have also been reported by prospective studies in which both fecal detection of *enteroviruses* and antibody seroconversion following *enterovirus* exposure were associated with CeD [20]. Kahrs et al. investigated serial stool samples collected from genetically susceptible children before the development of CeD autoantibodies. *Enterovirus* infection was more frequent among children who subsequently developed CeD, particularly when infections occurred after gluten introduction; higher viral loads and prolonged infections were associated with higher risk estimates [19]. Nevertheless, the available evidence is not entirely consistent. In the DIABIMMUNE cohort, Simre et al. found no marked overall differences in laboratory-confirmed common viral infections between children who subsequently developed CeD and matched controls [21]. Taken together, these longitudinal observations support a temporal association between selected early-life *enterovirus* or *parechovirus* exposures and subsequent CeD autoimmunity in some cohorts, but the results remain heterogeneous and, because the studies are observational, they do not establish causality [69].

#### 3.4.3. Cross-Sectional Virome and Phageome Evidence

Cross-sectional studies provide a different type of evidence, describing associations between viral markers or virome composition and established CeD rather than determining whether viral alterations precede disease onset.

In 2017, a study conducted by Vorobjova et al. aimed to investigate whether *enteroviruses* and immunoregulatory cells were associated with intestinal permeability in patients with CeD alone and in those with coexistent Type 1 Diabetes Mellitus (T1DM). CeD was associated with an increase in the levels of circulating zonulin (a tight junction regulator which plays a specific role in intestinal barrier function), which was correlated with *enterovirus* density in CeD patients, especially in those with severe atrophy in the small bowel mucosa and with concomitant T1DM. CeD was also characterized by the close relationship between the density of glutamic acid decarboxylase 65-positive epithelial cells and the *enterovirus*, zonulin levels and indoleamine 2,3-dioxygenase-positive dendritic cells [22]. Additional immunological evidence supports a possible relationship between enteroviral exposure and mucosal immune activation. In a subsequent study, Vorobjova et al. found that several circulating cytokines and chemokines were increased in children with CeD and that interleukin (IL)-17F, Interferon gamma-induced Protein 10 (IP-10), soluble tumor necrosis factor receptor type II (sTNFRII), monocyte chemoattractant protein 1 (MCP-1), and granulocyte–macrophage colony-stimulating factor (GM-CSF) correlated with the density of *enterovirus*-positive cells in the small bowel mucosa. These findings are consistent with an association between enteroviral exposure and Th1/Th17-skewed inflammatory responses, although the cross-sectional design does not establish a causal relationship [70].

A separate line of cross-sectional evidence derives from metagenomic characterization of the DNA virome and phageome. In 2022, a study conducted by El Mouzan et al. analyzed the virome of 40 children with new-onset CeD by shotgun metagenomic sequencing of mucosal and stool samples. Fecal and mucosal samples showed different viral profiles, and CeD was associated with compositional differences involving *Betapolyomavirus secuhominis* and the enterobacterial phages mEpX1 and mEpX2. A lower relative abundance of *Lactococcus* phage ul36 led the authors to propose a potentially protective association; however, this interpretation remains speculative and lacks functional validation. Because the study was cross-sectional, these findings cannot establish whether the observed virome/phageome differences preceded CeD, resulted from active disease or mucosal damage, or were influenced by other clinical or environmental factors [23].

Overall, the different lines of evidence should not be regarded as equivalent. Prospective studies provide temporal evidence linking selected early-life *enterovirus* exposures with later CeD autoimmunity, although causality remains unproven. Experimental studies provide mechanistic plausibility, whereas cross-sectional virome and phageome studies currently identify disease-associated viral signatures without establishing their temporal or causal role. Table 1 summarizes the principal human observational studies according to study design and temporal characteristics.

These observations support the idea that the intestinal virome acts as a modulator of mucosal immunity and may represent an environmental cofactor in the pathogenesis of CeD. Several recent reviews have consolidated this hypothesis, highlighting that the interaction between enteric viruses, infant immune maturation, timing of gluten introduction and its amount may contribute to disease development in genetically predisposed individuals [71,72,73]. Lerner et al. emphasized the potential role of the gut virome in the development of CeD and highlighted the need for further research to identify strategies that could restore a healthy microbiome, eliminate pathogenic microbes or enhance microbial resistance against viral pathogens [74].

## 4. From Colonization to Disease: The Gut Mycobiome in the Celiac Context

The term mycobiome refers to the fungal component of the microbiome in the human body [75]. Fungi represent a relatively small fraction of the total human microbiome, accounting for less than 0.1% of microbial cells; nevertheless, they contribute disproportionately to ecosystem diversity and function [76,77]. The human mycobiota is primarily found on mucosal and epithelial surfaces, including the skin, oropharynx, and gastrointestinal and genitourinary tracts [78]. In the human body, fungi can behave as commensals, maintaining a balanced interaction with the host, or as symbionts that contribute to metabolic and immune processes [79]. However, under conditions of immune imbalance or environmental perturbation, certain fungal species may act as opportunistic pathogens, contributing to local or systemic disease [80,81].

### 4.1. Composition of the Gut Mycobiome

Up to 99% of all gut microbial genes are of bacterial origin; therefore, fungi represent only a minor fraction of the resident gut microbiome [82]. Recent shotgun metagenomic sequencing analyses have revealed that fungi make up nearly 0.1% of the total microbes in the gut [82]. Their composition is highly dynamic, and it is shaped by numerous environmental factors.

In contrast to the bacteriome, overall fungal diversity is relatively low within the human host but is more variable between different individuals or even between different samples from the same person [78]. There is no consensus on the definition of a “healthy” gut mycobiome due to a variety of factors, such as the paucity and diversity of fungi in the gut and the temporary instability of the gut mycobiota during developmental periods [79]. However, gut fungal communities have been analyzed in many studies [83]; most gut mycobiome studies report a predominance of *Ascomycota* and *Basidiomycota*. Earlier literature also referred to *Zygomycota*; however, this polyphyletic historical grouping is no longer recognized, and its former members have been redistributed among several phylogenetically defined phyla, including *Mucoromycota* and *Zoopagomycota* [84]. A recent review by Hallen-Adams and colleagues identified potential fungal species inhabiting the intestinal niche, belonginpag to the genera *Candida*, *Cryptococcus*, *Malassezia*, *Aspergillus*, *Saccharomyces*, *Galactomyces*, *Trichosporon*, and *Cladosporium* [79].

Recently, Nash and colleagues (2017) analyzed 317 stool samples from a healthy cohort by Internal Transcribed Spacer 2 (ITS2) region and *18S* rRNA gene sequencing within the Human Microbiome Project (HMP). The authors reported that the intestinal mycobiota is primarily dominated by *Malassezia*, *Candida* and *Saccharomyces*, with *S. cerevisiae*, *M. restricta* and *C. albicans* identified in 96.8%, 88.3%, and 80.8% of samples, respectively [78].

### 4.2. Fungal Intestinal Colonization

Fungal colonization of the human gut begins remarkably early in life and follows a strongly dynamic trajectory during the first years of development, influenced by maternal, environmental, dietary, and immunological factors [85]. For many years, the possibility of “in utero” microbial colonization (including the presence of fungal elements) has been intensely debated. Although some studies have reported microbial DNA or signatures in placental tissues, amniotic fluid or meconium, more recent and methodologically rigorous analyses suggest that these signals likely reflect environmental or reagent contamination rather than the existence of a stable prenatal microbiome. This perspective has led to the consensus that the fetal gut is essentially sterile and that colonization begins predominantly at birth, when exposure to maternal and environmental microbes becomes possible [86]. In newborns, *Candida*, *Malassezia*, and *Saccharomyces* are prevalent.

Gut mycobiome composition is strongly influenced by the mode of birth: vaginal delivery promotes vertical transmission of *Candida* and other maternal fungal species, while cesarean delivery is associated with a more skin-like mycobiome profile, with lower diversity, suggesting that the initial exposure routes exert lasting effects on fungal colonization patterns [87].

Breast milk introduces fungi such as *Candida* and *Malassezia*, but the presence of immunoglobulins and oligosaccharides limits excessive fungal growth. With the introduction of solid foods during weaning, the mycobiome undergoes enrichment and diversification. Dietary and environmental exposures introduce new fungal taxa, including *Saccharomyces*, *Debaryomyces*, *Penicillium*, and *Aspergillus*, which diversify the ecological landscape of the gut [80,88]. This stage is also characterized by the strengthening of interkingdom interactions, particularly fungal–bacterial crosstalk, which plays a central role in shaping mucosal immune system maturation and barrier function. These interactions include competition for nutrients, the modulation of metabolic outputs and reciprocal regulation of immune responses, illustrating how the gut mycobiome contributes to the broader microbial ecosystem [89].

From school-age through adulthood, the gut mycobiome gradually stabilizes, typically becoming dominated by *Candida*, *Saccharomyces*, and *Malassezia*. Nevertheless, it remains more variable and less resilient than the bacterial microbiome, showing substantial fluctuations in response to external factors. Across the lifespan, diet, antibiotic exposure, lifestyle, environmental contacts, and host immune status continue to modulate both fungal diversity and richness, sometimes leading to transient dysbiosis [78,90,91].

### 4.3. Mycobiome in Health and Disease

The gut mycobiome plays an important role in maintaining intestinal homeostasis through continuous interactions with bacterial communities and the host immune system [17]. Although fungi represent only a minor component of the gut microbiome, they actively modulate immune responses, epithelial barrier function, and microbial ecology.

Gut mycobiome dysbiosis has been associated with chronic inflammatory disorders, including autoimmune [92], metabolic [93], neurological [94], neoplastic diseases [95] and inflammatory bowel disease, where increased abundance of *Candida* spp. and other opportunistic fungi has been consistently reported [75,90]. These observations suggest that fungal alterations may contribute to chronic intestinal inflammation and provide a biological basis for investigating their role in celiac disease.

### 4.4. Cross-Sectional Mycobiome Evidence in CeD

CeD-specific human mycobiome evidence is currently limited and derives predominantly from cross-sectional analyses. These studies can identify disease- or genotype-associated fungal compositional differences but cannot determine whether such alterations precede disease onset or result from CeD, dietary changes, intestinal inflammation, or other confounding factors.

A recent study analyzed the gut mycobiome in children with newly diagnosed CeD to investigate possible associations between gut fungi and this disease. Intestinal mucus and stool samples were collected from children with CeD and from controls and analyzed by ITS-based fungal sequencing. The results showed that in CeD children, *Tricholomataceae*, *Saccharomycetaceae*, *Saccharomycetes*, *Saccharomyces cerevisiae*, and *Candida* were relatively more abundant, while *Pichiaceae*, *Pichia kudriavzevii*, *Pneumocystis*, and *Pneumocystis jirovecii* were relatively less abundant. Overall diversity (alpha and beta) did not differ significantly between celiac patients and controls, although mucus and stool samples displayed distinct profiles. The study therefore identified CeD-associated fungal compositional differences, whose functional and pathogenetic significance remains uncertain and requires longitudinal and mechanistic investigation [24].

Recent evidence has begun to indicate an association between host genetics and microbial composition, providing new insights into disease susceptibility. A 2026 study by Salamon et al. investigated gut bacterial and fungal signatures in CeD children, their siblings and controls in relation to HLA-DQ2/DQ8 status. Distinct microbial profiles were associated with both disease status and host genetic susceptibility, and fungal signatures were also detected in genetically susceptible siblings without overt CeD. However, because the study was cross-sectional and lacked longitudinal follow-up, these findings cannot establish that the fungal signatures preceded disease development or predict future CeD. Their temporal and potential clinical significance therefore requires prospective longitudinal validation [25].

Complementary immunological evidence also supports a possible relationship between fungal exposure and CeD. Sendid et al. investigated antibody responses against two distinct molecular targets associated with *Candida albicans*: anti-*Saccharomyces cerevisiae* antibodies (ASCA), which recognize oligomannosidic epitopes that can be expressed by *Candida albicans*, and antibodies against hyphal wall protein 1 (Hwp1), a protein expressed during the invasive hyphal phase and characterized by peptide sequence homologies with gliadin. The authors observed a significant correlation between ASCA and anti-Hwp1 responses in patients with CeD, reinforcing the hypothesis that exposure to *Candida albicans* may be associated with CeD-related immune responses and raising the possibility of antigenic or molecular mimicry. However, these serological findings do not demonstrate intestinal fungal dysbiosis or establish a causal role for *Candida albicans* in disease development [96].

A subsequent predictive analysis by El Mouzan et al. evaluated microbial DNA from bacterial, viral, and fungal communities in fecal and mucosal samples from 40 children with CeD and 39 controls to assess the ability of microbiota signatures to discriminate between the two groups. The fungal component showed only modest discriminatory performance in fecal samples (Area Under the Curve, AUC, 67.7%) and poor performance in mucosal samples (AUC 35%), while combined bacterial and viral signatures performed better. These findings suggest that, despite detectable fungal compositional alterations, current fungal signatures alone have limited diagnostic value and require validation before being considered clinically useful biomarkers [97].

The principal human studies investigating gut fungal alterations in CeD are summarized in Table 2.

## 5. Impact of a Gluten-Free Diet on the Gut Virome and Mycobiome

Although the gluten-free diet (GFD) represents the cornerstone of CeD management, its effects on the gut virome and mycobiome remain largely unknown. To date, direct longitudinal studies evaluating these microbial components in patients with CeD are lacking. The available evidence on the gut virome derives mainly from a longitudinal metagenomic study performed in healthy adults, in which Garmaeva et al. observed that the gut virome remained relatively stable over time but could be partially modulated by dietary intervention, particularly in individuals with low baseline viral diversity. Therefore, these findings should not be directly extrapolated to patients with CeD but rather considered indirect evidence that dietary interventions may influence viral community structure [98].

These findings suggest that one possible mechanism is that dietary effects on the virome may be indirectly mediated by alterations in the bacterial microbiome, which represents the primary host community for bacteriophages. Indeed, previous studies have shown that dietary changes can rapidly influence bacteriome composition and metabolic activity, thereby secondarily affecting phage populations and viral community structure [99].

While the effect of a GFD on the virome is better known, its impact on the gut mycobiome is poorly understood, and direct longitudinal studies are currently lacking. However, given that fungal communities are highly sensitive to dietary inputs, it is plausible that gluten withdrawal may alter mycobiome composition. Diet-driven changes in carbohydrate availability, fiber intake and the consumption of fermented foods may influence the abundance of key fungal taxa such as *Saccharomyces* and *Candida*, which are commonly detected in the human gut [79].

**Figure 2:**
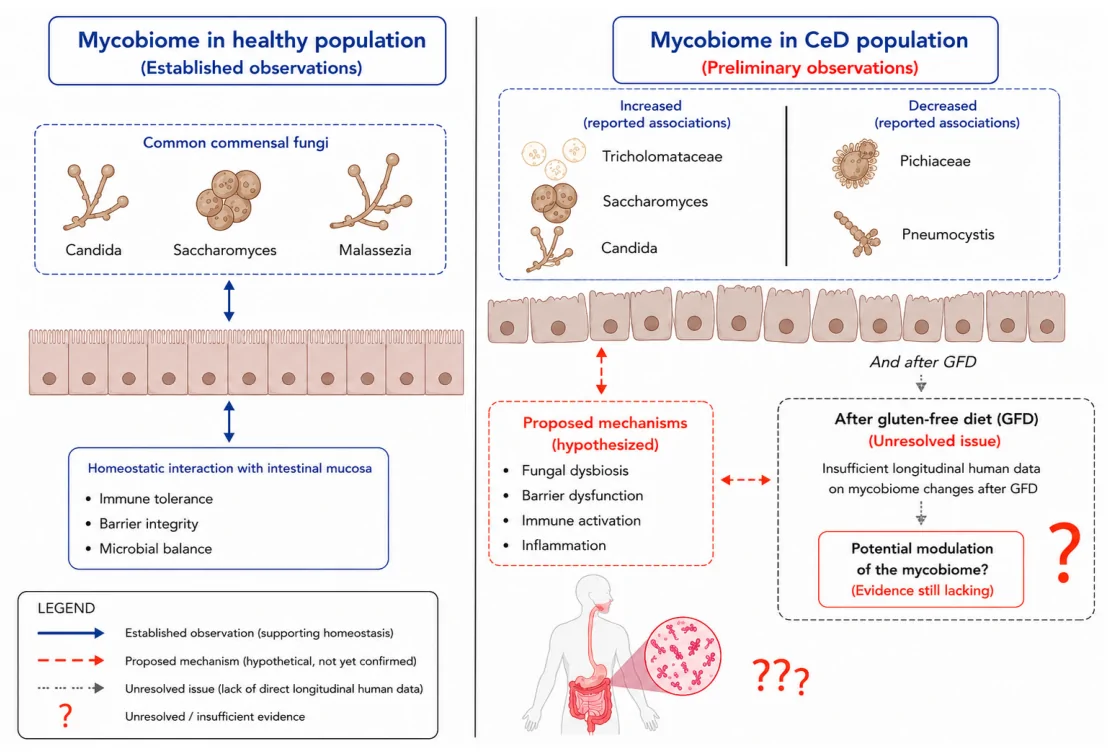
Current evidence and proposed interactions between the gut mycobiome, celiac disease and the gluten-free diet. Solid arrows indicate established observations, dashed arrows proposed mechanisms, and dotted arrows unresolved relationships. CeD: Celiac Disease; GFD: Gluten-Free Diet.

Notably, available evidence suggests that a GFD does not fully restore a “healthy” microbial profile in patients with CeD. While bacterial dysbiosis may partially improve, some alterations persist despite strict adherence to a GFD, raising the possibility that fungal imbalances may similarly be incompletely reversible or independent of gluten exposure [100]. Overall, these observations highlight a critical gap in current knowledge regarding the ecological impact of gluten exclusion beyond the bacterial microbiome. Future longitudinal and multi-omics studies integrating virome, mycobiome, and bacteriome analyses are needed to determine whether dietary interventions can restore microbial homeostasis or whether persistent alterations contribute to ongoing immune dysregulation in treated CeD (Figure 2).

## 6. Limitations

Interpretation of the available evidence on the gut virome and mycobiome in CeD requires careful consideration of several methodological limitations [30,101]. Virome studies are challenged by the large proportion of unclassified viral sequences, commonly referred to as viral dark matter [55], reflecting the incompleteness of current viral reference databases and limiting taxonomic assignment [102]. In addition, distinguishing integrated prophages from actively replicating bacteriophages remains technically difficult, potentially affecting the interpretation of phage dynamics [103]. The choice of sequencing strategy also influences study findings: bulk shotgun metagenomics enables the simultaneous characterization of multiple microbial components but can provide limited viral coverage and reduced sensitivity for low-abundance viral genomes compared with virus-like particle-enriched approaches [104].

Similar limitations affect mycobiome research [101]. Fungal DNA represents only a very small fraction of the total microbial DNA within intestinal samples, making fungal community profiling particularly susceptible to reagent and environmental contamination [101,105]. Furthermore, many fungal taxa detected in fecal samples may originate from recent dietary intake rather than stable gut colonization, complicating the distinction between resident and transient organisms [106]. Amplicon-based studies targeting the internal transcribed spacer (ITS) regions are also subject to primer-related amplification bias, which may influence the relative abundance of specific taxa [107]. Finally, microbiome sequencing data are intrinsically compositional, meaning that apparent changes in the relative abundance of one microorganism may reflect changes in other community members rather than true biological expansion or depletion [108].

Interpretation of the available evidence should consider several potential confounding factors, including age, geographical origin, delivery mode, breastfeeding, antibiotic exposure, previous viral infections, dietary habits, disease activity, serological status, degree of mucosal damage, HLA genotype, adherence to a GFD, and the type of biological sample analyzed, such as feces or duodenal biopsies [18,30,101,109,110]. These variables differ substantially among studies and may partly explain the heterogeneity of published findings. Together, these methodological challenges contribute substantially to the heterogeneity of published studies and currently limit direct comparisons across cohorts.

An additional limitation is that, because this was a narrative review, no validated risk-of-bias instrument, evidence-grading framework, scoring system, or predefined quantitative rubric was applied.

## 7. Conclusions

While the genetic basis of CeD and the central role of gluten exposure are universally recognized, growing evidence suggests that additional microbial factors, including early-life viral exposures and fungal compositional alterations, may be associated with CeD-related immune and microbial changes. However, the currently available evidence remains limited, is largely observational, and does not establish causal relationships.

Overall, the strongest human evidence currently supports an association between repeated early-life *enterovirus* exposure and the subsequent development of CeD autoimmunity in genetically susceptible children. In contrast, evidence regarding alterations of the gut phageome and mycobiome remains limited, largely cross-sectional and insufficient to establish causality. Future longitudinal multi-omics studies integrating virome, mycobiome, bacteriome and host immune responses will be essential to clarify whether these microbial alterations represent disease drivers, modifiers or secondary consequences of intestinal inflammation.

The impact of the GFD on the gut virome and mycobiome also remains poorly understood. Current evidence regarding virome changes after dietary intervention derives primarily from studies performed in healthy adults rather than in patients with CeD, whereas longitudinal mycobiome studies in CeD are currently unavailable. Consequently, it remains uncertain whether gluten withdrawal restores these microbial ecosystems or whether persistent alterations contribute to ongoing immune dysregulation.

Future longitudinal, multicenter, multi-omics studies integrating bacterial, viral and fungal communities together with host immune and genetic data will be essential to clarify temporal relationships and identify biologically relevant microbial signatures. At present, modulation of the gut virome or mycobiome should be regarded as a promising area for future research rather than an established therapeutic strategy for CeD.

This review highlights the emerging role of the intestinal virome and mycobiome as potential environmental cofactors in the pathogenesis of CeD. These findings identify potential avenues for future experimental research; however, no preventive or therapeutic implications can currently be drawn from the available evidence.

## Figures and Tables

**Table 1:** Summary of the principal human studies investigating viral exposure and the intestinal virome/phageome in CeD, grouped according to study design and temporal characteristics.

Reference	Study Design	Population (*N*)	Control/Comparator Group	Biological Specimen	Detection Method	Follow-Up/Temporal Assessment	Main Findings	Main Limitations
TEDDY study (Lindfors et al., 2020) [18]	Prospective multicenter birth cohort	Genetically susceptible children enrolled in the TEDDY cohort (*N* = 8676); 83 children who developed CeD autoimmunity were included in the nested analysis	Matched children without CeD autoimmunity (*N* = 83)	Serial stool samples	*Enterovirus* PCR/metagenomics	Enrollment before 4.5 months of age for a 15-year follow-up with annual screening	Repeated *enterovirus* exposure during the first two years of life was associated with an increased risk of celiac disease autoimmunity, particularly in children with higher gluten intake, supporting a virus–gluten “double-hit” hypothesis.	Observational design; restricted to genetically susceptible children; residual confounding cannot be excluded.
Kahrs et al., 2019 [19]	Nested case–control within prospective birth cohort	Genetically susceptible children (HLA-DR3-DQ2/DR4-DQ8); CeD cases (*N* = 25)	Matched controls (*N* = 50)	Serial stool samples and serum	RT-PCR for *enterovirus/adenovirus*; serology for CeD autoantibodies	Monthly sampling from 3–36 months; follow-up ~9.9 years	*Enterovirus* infection before CeD autoimmunity was significantly more frequent in cases, especially after gluten introduction; stronger association with high viral load and prolonged infection. No association with *adenovirus*.	Limited number of cases; genetically selected cohort; residual confounding; observational design.
Oikarinen et al., 2021 [20]	Prospective birth cohort	Children from a longitudinal birth cohort (*N* = 41)	Control children matched for calendar month and year of birth (+/−2 months), sex, city of residency and HLA-DQ alleles (*N* = 53)	Stool samples and serum	*Enterovirus* RT-PCR and serology	From birth to CeD seroconversion, with blood samples screened every 3–12 months	*Enterovirus* infection and seroconversion preceded the onset of celiac disease, supporting a temporal association between viral exposure and disease development.	Limited number of incident CeD cases; observational design; causality cannot be established.
Simre et al., 2019 [21]	Case–control (DIABIMMUNE cohort)	Children newly diagnosed with CeD (*N* = 29)	Matched controls (*N* = 29)	Stool, nasal swabs, serum	Viral PCR and serology for common infections	Early infancy sampling (3–6 months); assessment at diagnosis	No major differences in overall viral infections between groups; slight increase in *rhinovirus* detection in CeD cases, but no consistent serological differences.	Small sample size; heterogeneous cohorts; limited sampling window; low power for individual viruses.
Vorobjova et al., 2017 [22]	Observational, cross-sectional	Patients with CeD with (*N* = 23) or without T1DM (N = 15)	Patients with functional dyspepsia and normal small bowel mucosa (*N* = 40)	Serum and duodenal biopsies	*Enterovirus* immunohistochemistry; immunological markers (zonulin, IDO, GAD65)	Time of endoscopy assessment; no follow-up available	Increased circulating zonulin levels correlated with *enterovirus* density, particularly in patients with severe villous atrophy and concomitant T1DM, suggesting impaired intestinal barrier function and immune dysregulation.	Small sample size; cross-sectional design; no causal inference; limited adjustment for confounders.
El Mouzan et al., 2022 [23]	Case–control	Children with newly diagnosed CeD (*N* = 40)	Healthy children (*N* = 39)	Stool and duodenal mucosa	Shotgun metagenomic sequencing	Time of endoscopy assessment; no follow-up available	CeD was associated with altered viral composition, including increased relative abundance of human *polyomavirus 2 (JC polyomavirus*; current ICTV species *Betapolyomavirus secuhominis*) and enterobacterial phages and reduced relative abundance of *Lactococcus* phage ul36, which was hypothesized to have a potentially protective association.	Small pediatric cohort; cross-sectional design; no functional validation; no assessment of causality.

CeD: Celiac Disease; HLA: Human Leukocyte Antigen; ICTV: International Committee on Taxonomy of Viruses; T1DM: Type 1 Diabetes Mellitus.

**Table 2:** Summary of key human studies reporting gut mycobiome associations in CeD.

Reference	Study Design	Population (*N*)	Control/Comparator Group	Biological Specimen	Detection Method	Follow-Up/Temporal Assessment	Main Findings	Main Limitations
El Mouzan et al., 2022 [24]	Case–control	Children with newly diagnosed CeD (*N* = 40)	Healthy children (*N* = 39)	Stool and duodenal mucus	ITS-based fungal sequencing	Sample collection was performed while participants were consuming a gluten-containing diet; no follow-up was available	CeD was associated with an altered fungal composition, including increased relative abundance of *Saccharomyces cerevisiae*, *Candida* spp., *Tricholomataceae*, *Saccharomycetaceae* and lower relative abundance of *Pichia kudriavzevii* and *Pneumocystis jirovecii*. No significant differences in α- or β-diversity were observed.	Single-center pediatric cohort; cross-sectional design; absence of functional analyses; limited sample size; no causal inference.
Salamon et al., 2026 [25]	Cross-sectional observational	Children with newly diagnosed CeD (*N* = 14)	Unaffected siblings of CeD patients (*N* = 16) and healthy controls (*N* = 19)	Stool samples	Shotgun metagenomic sequencing	Time of CeD diagnosis; no follow-up available	Host HLA-DQ2/DQ8 genotype was associated with distinct fungal community profiles. Specific fungal signatures were also identified in genetically susceptible siblings without CeD.	Cross-sectional design; relatively small cohort; findings require external validation; no mechanistic evidence demonstrating causality.

CeD: Celiac Disease; ITS: Internal Transcribed Spacer.

## Data Availability

No new data were created or analyzed in this study. Data sharing is not applicable to this article.

## Supplementary Materials

The following supporting information can be downloaded at: https://www.mdpi.com/article/10.3390/nu18162721/s1.

## Abbreviations

The following abbreviations are used in this manuscript:

ASCA	Anti-Saccharomyces Cerevisiae Antibodies
CeD	Celiac Disease
DNA	DeoxyriboNucleic Acid
GFD	Gluten-Free Diet
GM-CSF	Granulocyte–Macrophage Colony-Stimulating Factor
HCV	Hepatitis C Virus
HLA	Human Leucocyte Antigen
HMP	Human Microbiome Project
HWP1	Hyphal wall protein 1
ICTV	International Committee on Taxonomy of Viruses
IFN	Interferon
IL	Interleukine
ITS	Internal Transcribed Spacer
ITS2	Internal Transcribed Spacer 2
MCP-1	Monocyte Chemoattractant Protein 1
NK	Natural Killer
RNA	RiboNucleic Acid
sTNFRII	soluble Tumor Necrosis Factor Receptor type II
T1DM	Type 1 Diabetes Mellitus
TH1	T helper 1
Treg	Regulatory T Cell
