# Supplementary material for: Beyond Bacteria: The Emerging Role of the Gut Virome and Mycobiome in Celiac Disease
Source: Nutrients. 2026 Aug 20;18(16):2721. doi: 10.3390/nu18162721 (PMC13516525; doi:10.3390/nu18162721)
Supplement: Supplementary file 1 [file nutrients-18-02721-s001.zip › nutrients-4440329-supplementary.pdf]

Supplementary Table S1. Database-specific search strategies used to retrieve potentially eligible records.

	Virome	Mycobiome
PubMed	("Celiac Disease"[MeSH Terms] OR "celiac disease"[Title/Abstract] OR "coeliac disease"[Title/Abstract] OR celiac[Title/Abstract] OR coeliac[Title/Abstract]) AND ("Virome"[MeSH Terms] OR virome[Title/Abstract] OR "gut virome"[Title/Abstract] OR "intestinal virome"[Title/Abstract] OR "viral microbiome"[Title/Abstract] OR enterovirus*[Title/Abstract] OR parechovirus*[Title/Abstract] OR reovirus*[Title/Abstract] OR "Bacteriophages"[MeSH Terms] OR bacteriophage*[Title/Abstract] OR phage*[Title/Abstract] OR phageome[Title/Abstract])	("Celiac Disease"[MeSH Terms] OR "celiac disease"[Title/Abstract] OR "coeliac disease"[Title/Abstract] OR celiac[Title/Abstract] OR coeliac[Title/Abstract]) AND ("Mycobiome"[MeSH Terms] OR mycobiome[Title/Abstract] OR "gut mycobiome"[Title/Abstract] OR "intestinal mycobiome"[Title/Abstract] OR "fungal microbiome"[Title/Abstract] OR mycobiota[Title/Abstract] OR "fungal microbiota"[Title/Abstract] OR "fungal community"[Title/Abstract] OR "fungal communities"[Title/Abstract] OR "Fungi"[MeSH Terms] OR fungi[Title/Abstract] OR fungal[Title/Abstract])
Scopus	TITLE-ABS-KEY("celiac disease" OR "coeliac disease" OR celiac OR coeliac) AND TITLE-ABS-KEY(virome OR "gut virome" OR "intestinal virome" OR "viral microbiome" OR enterovirus* OR parechovirus* OR reovirus* OR bacteriophage* OR phage* OR phageome)	TITLE-ABS-KEY("celiac disease" OR "coeliac disease" OR celiac OR coeliac) AND TITLE-ABS-KEY(mycobiome OR "gut mycobiome" OR "intestinal mycobiome" OR "fungal microbiome" OR mycobiota OR "fungal microbiota" OR "fungal community" OR "fungal communities" OR fungi OR fungal)
Web of Science Core Collection	("celiac disease" OR "coeliac disease" OR celiac OR coeliac) AND (virome OR "gut virome" OR "intestinal virome" OR "viral microbiome" OR enterovirus* OR parechovirus* OR reovirus* OR bacteriophage* OR phage* OR phageome)	("celiac disease" OR "coeliac disease" OR celiac OR coeliac) AND (mycobiome OR "gut mycobiome" OR "intestinal mycobiome" OR "fungal microbiome" OR mycobiota OR "fungal microbiota" OR "fungal community" OR "fungal communities" OR fungi OR fungal)
